# Supplementary material for: Impaired liver regeneration in aged mice can be rescued by silencing Hippo core kinases MST1 and MST2
Source: EMBO Mol Med. 2016 Dec 9;9(1):46–60. doi: 10.15252/emmm.201506089 (PMC5210079; doi:10.15252/emmm.201506089)
Supplement: Supplementary file 1 — Appendix [file EMMM-9-46-s001.pdf]

# Appendix

## Rescue of impaired liver regeneration in aged mice by silencing the Hippo pathway

Giulio Loforese[1], Thomas Malinka[1], Adrian Keogh[1], Felix Baier[1], Cedric Simillion[2], Thanos D. Halazonetis[3], Daniel Candinas[1] and Deborah Stroka[1]

Table of Contents

**Figure S1** MST1, LATS and MST2 protein expression during liver regeneration

**Figure S2** Validation of LATS1 and p-LATS1 antibody specificty by Western blot.

**Figure S3** RT-qPCR of regenerating mouse liver tissue sham, 6, 24 and 48 following PH

**Figure S4** Western blot detection of p-LATS in young and aged liver post PH

**Figure S5** Screening for siRNA sequences targeting the Hippo core kinases MST1 and MST2 in BNL mouse cell line.

**Figure S6** Pathway analysis of siMST versus siScr

Genomic microarray was performed on liver samples on day 3 following injection of either siMST or siScr. Pathway analysis was performed using the SetRank method.

**Figure S7** Heat maps of genes regulated by siMST compared to siScr

A Interferon gamma signalling
B extracellular matrix remodelling
C mitotic prometaphase

**Figure S8** Assessment of liver injury and off target effects from siRNA:liposomes

**Figure S9** Measurement of hepatocyte dimensions

**Figure S10** Serum transaminases (ALT and AST) and alkaline phosphatase (ALP) in regenerating aged livers ± MST KD (aged MST)

Appendix Figure S1

a

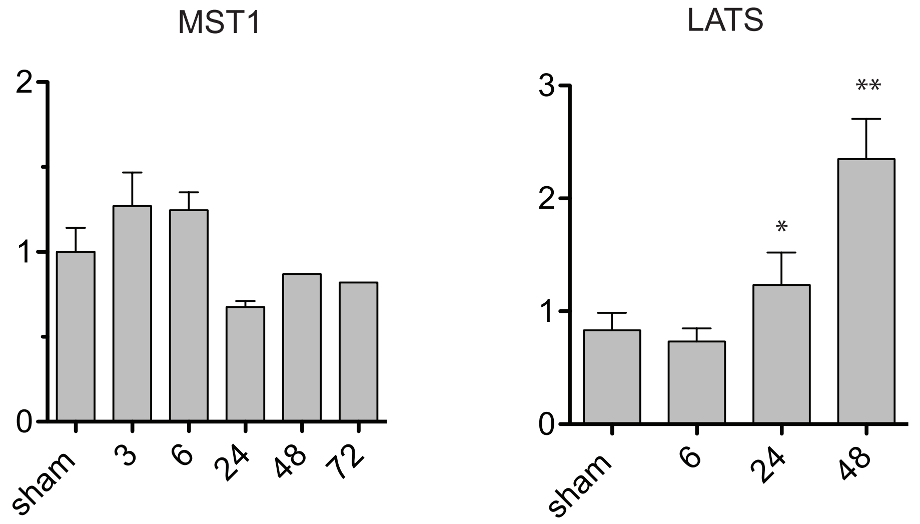


b

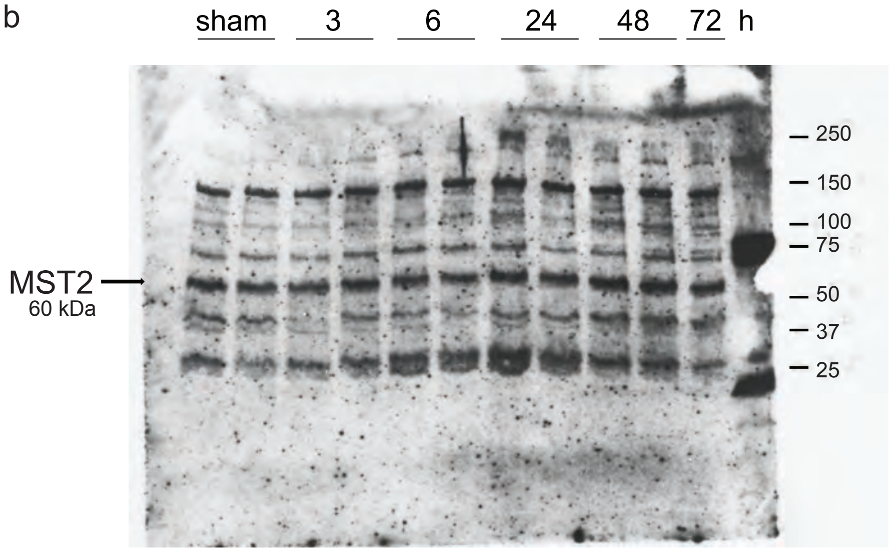


**Figure S1 MST1, LATS and MST2 protein expression during liver regeneration**

A MST and LATS total protein quantification from Figure 1B.
Data are presented as mean ± SD. p values were calculated with Student's t-test comparing values at each time point to sham operated control tissue compared to sham controls.

B MST2 Western blot membrane. MST2 migrates at approximately 60kDa.

Appendix Figure S2

anti-p-LATS (Ser909) 1:500, #9157 (Cell Signaling)

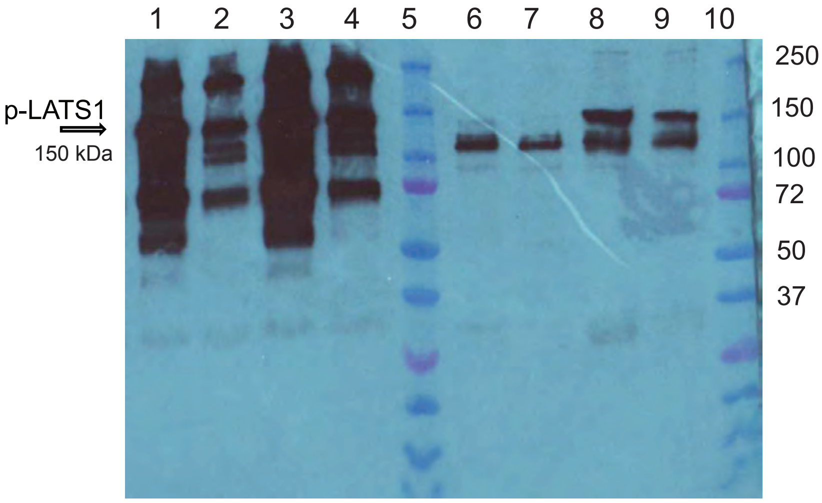


Lane 1: HeLa cells + TPA (5μg)
Lane 2: Hela cells (5μg)
Lane 3: HeLa cells (10μg)
Lane 4: HeLa cells + TPA (10μg)
Lane 5: MW Marker (BioRad)
Lane 6-7: Liver protein extract, control (20μg)
Lane 8-9: Liver protein extract, post PH (20μg)
Lane 10: MW Marker (BioRad)

anti-LATS 1:5000, ab70561 (Abcam)

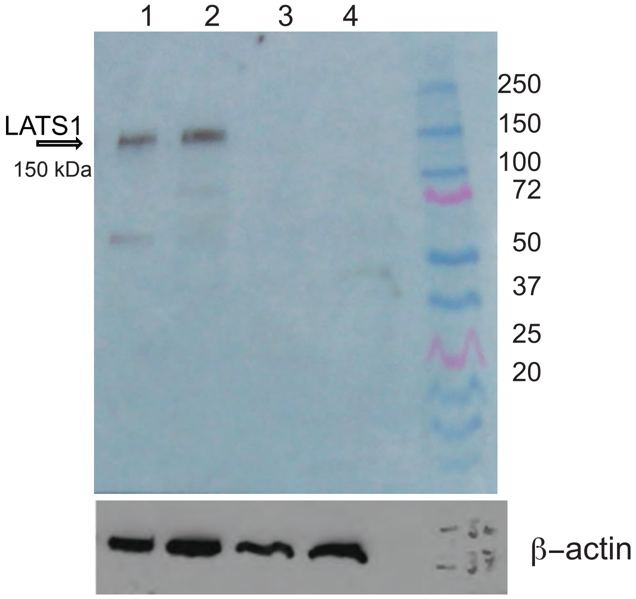


Lanes 1 & 2: Hep3B cells (10μg)
Lane 3 + LATS1siRNA 50pmole
Lane 4 + LATS1 siRNA 100pmole
Lane 4: MW Marker (BioRad)

**Figure S2 Validation of LATS1 and p-LATS1 antibody specificty by Western blot.**

Appendix Figure S3

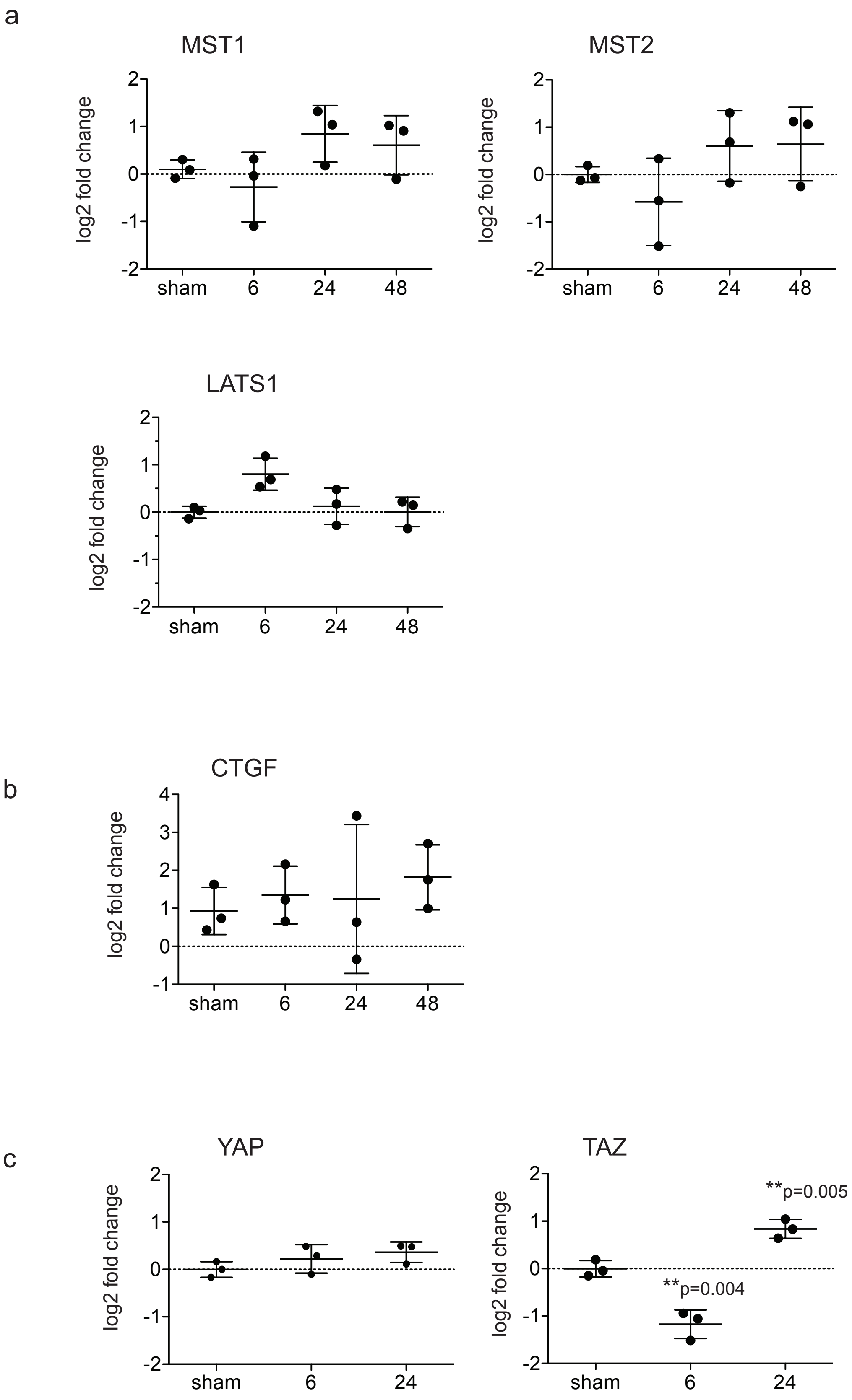


**Figure S3 RT-qPCR of regenerating mouse liver tissue sham, 6, 24 and 48 following PH**

Data are presented as mean ± SD. p values were calculated with a student's t-test and time points were compared to sham controls.

Appendix Figure S4

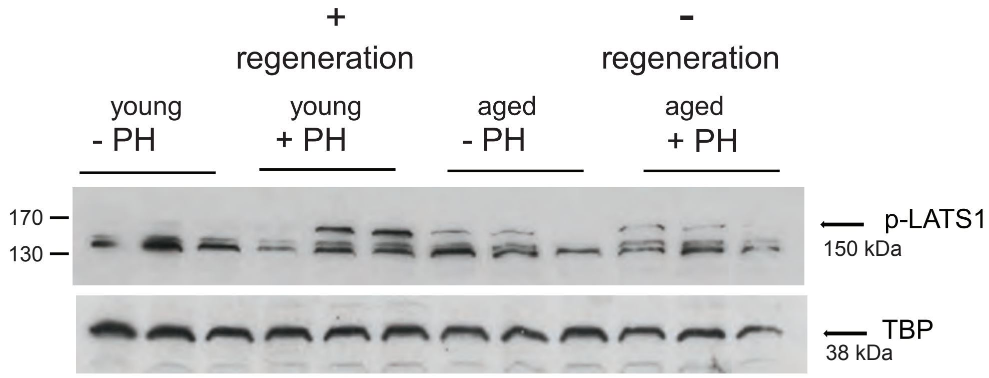


**Figure S4 Western blot detection of p-LATS in young and aged liver post PH**

Liver protein extracts were isolated from young and aged mice without (-) and with (+) a partial hepatectomy. At 40 h post PH, young mice were determined to be regenrating (+) and aged micewere non-regenerating (-). TBP was used as a loading control. Each band is from an independent animal.

Appendix Figure S5

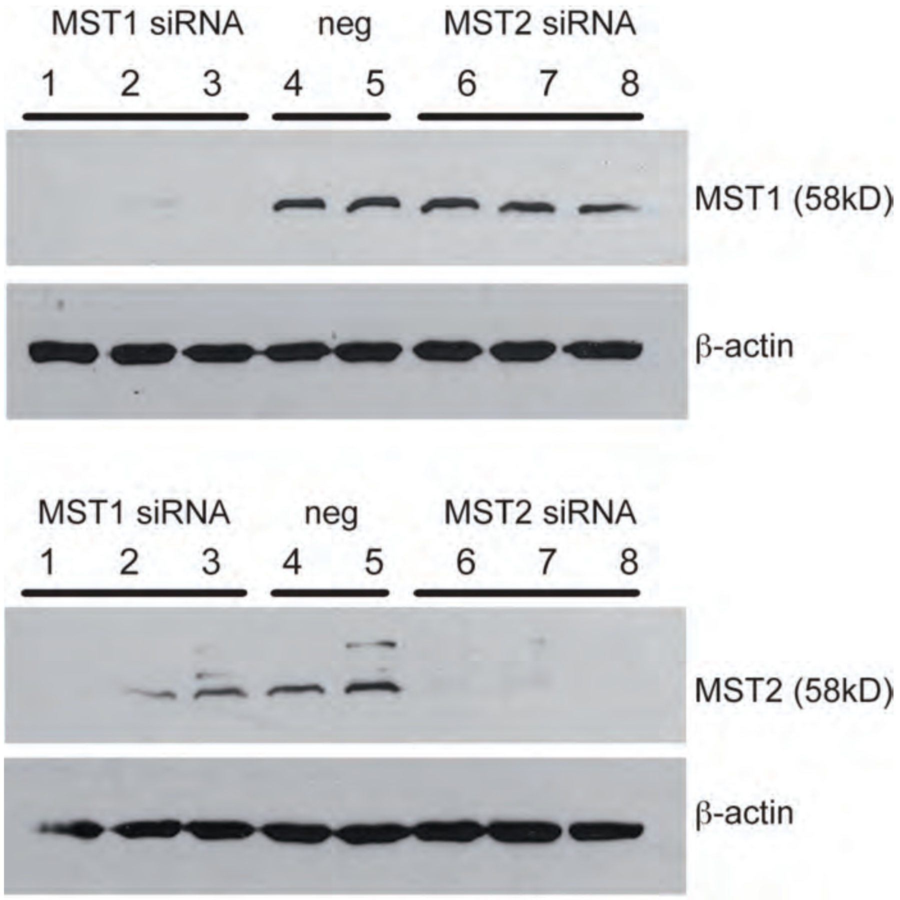


**Figure S5 Screening for siRNA sequences targeting the Hippo core kinases MST1 and MST2 in BNL mouse cell line.**

(A) MST1 and (B) MST2 protein levels analysed by western blot 24 h after siRNA transfection. β-actin was used as protein loading control.

Lane 1) MST1/STK4 sequence ID: s81598.
Lane 2) MST1/STK4 sequence ID: s81597
Lane 3) MST1/STK4 sequence ID: s81599
Lane 4) negative control sequence
Lane 5) negative control sequence
Lane 6) MST2/STK3 sequence ID:S211749
Lane 7) MST2/STK3 sequence ID:S211750
Lane 8) MST2/STK3 sequence ID:S211751

Appendix Figure S6

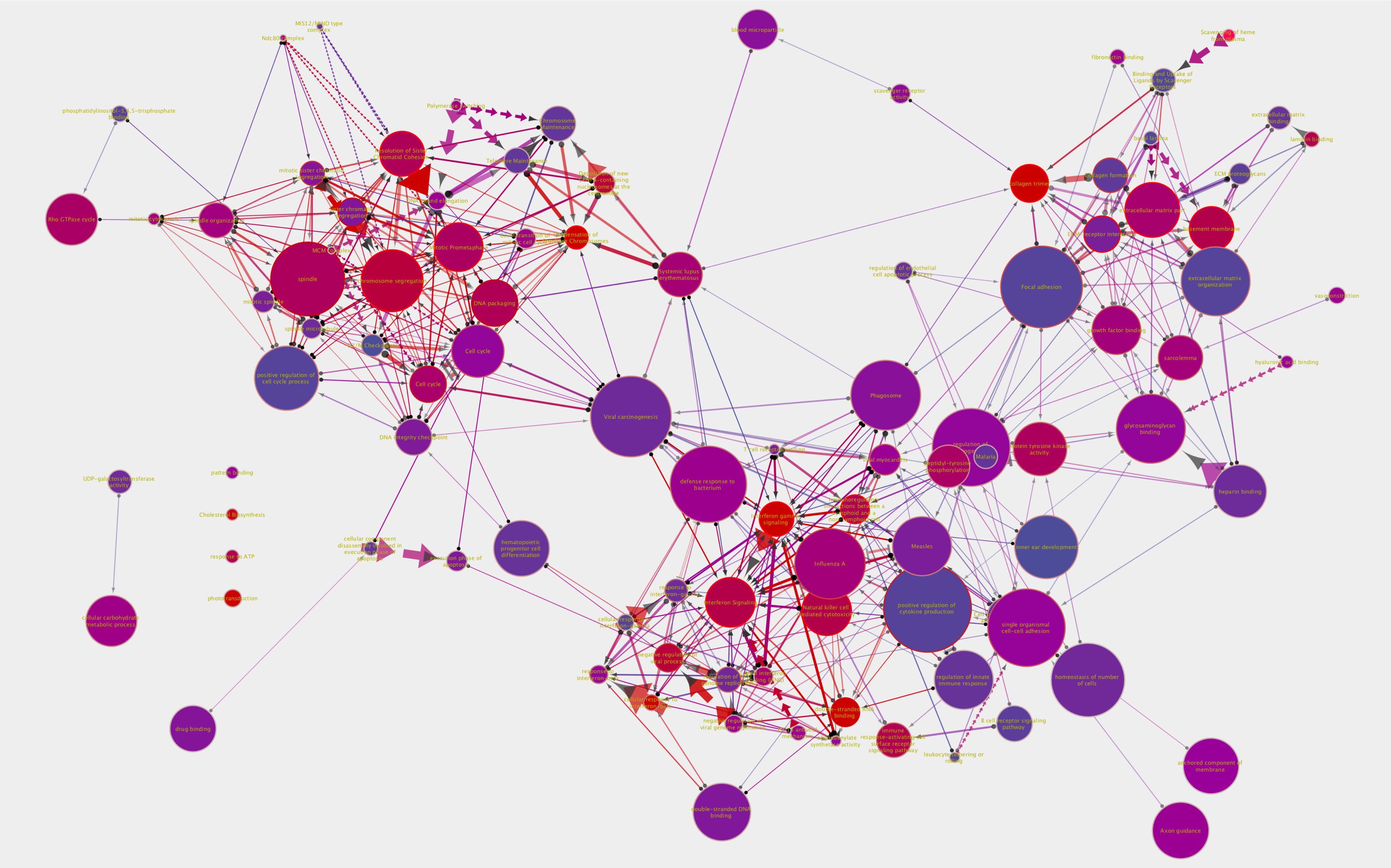

**Figure S6 – Pathway analysis of siMST versus siScr**

Genomic microarray was performed on liver samples on day 3 following injection of either siMST or siScr. Pathway analysis was performed using the SetRank method.

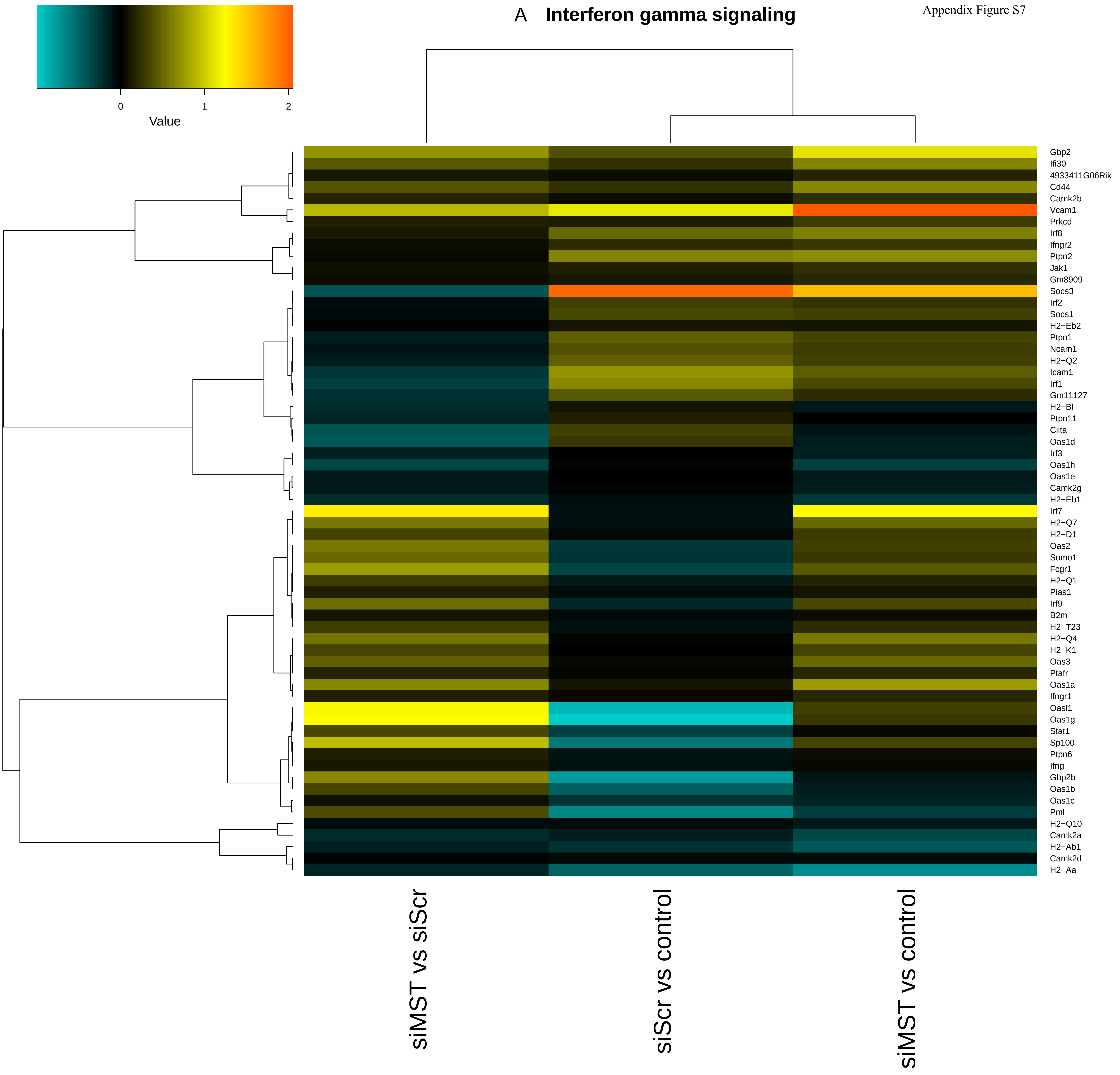
A Interferon gamma signaling
Value
0
1
2
Gbp2
Ifi30
4933411G06Rik
Cd44
Camk2b
Vcam1
Prkcd
Irf8
Ifngr2
Ptpn2
Jak1
Gm8909
Socs3
Irf2
Socs1
H2−Eb2
Ptpn1
Ncam1
H2−Q2
Icam1
Irf1
Gm11127
H2−Bl
Ptpn11
Ciita
Oas1d
Irf3
Oas1h
Oas1e
Camk2g
H2−Eb1
Irf7
H2−Q7
H2−D1
Oas2
Sumo1
Fcgr1
H2−Q1
Pias1
Irf9
B2m
H2−T23
H2−Q4
H2−K1
Oas3
Ptafr
Oas1a
Ifngr1
Oasl1
Oas1g
Stat1
Sp100
Ptpn6
Ifng
Gbp2b
Oas1b
Oas1c
Pml
H2−Q10
Camk2a
H2−Ab1
Camk2d
H2−Aa
siMST vs siScr
siScr vs control
siMST vs control

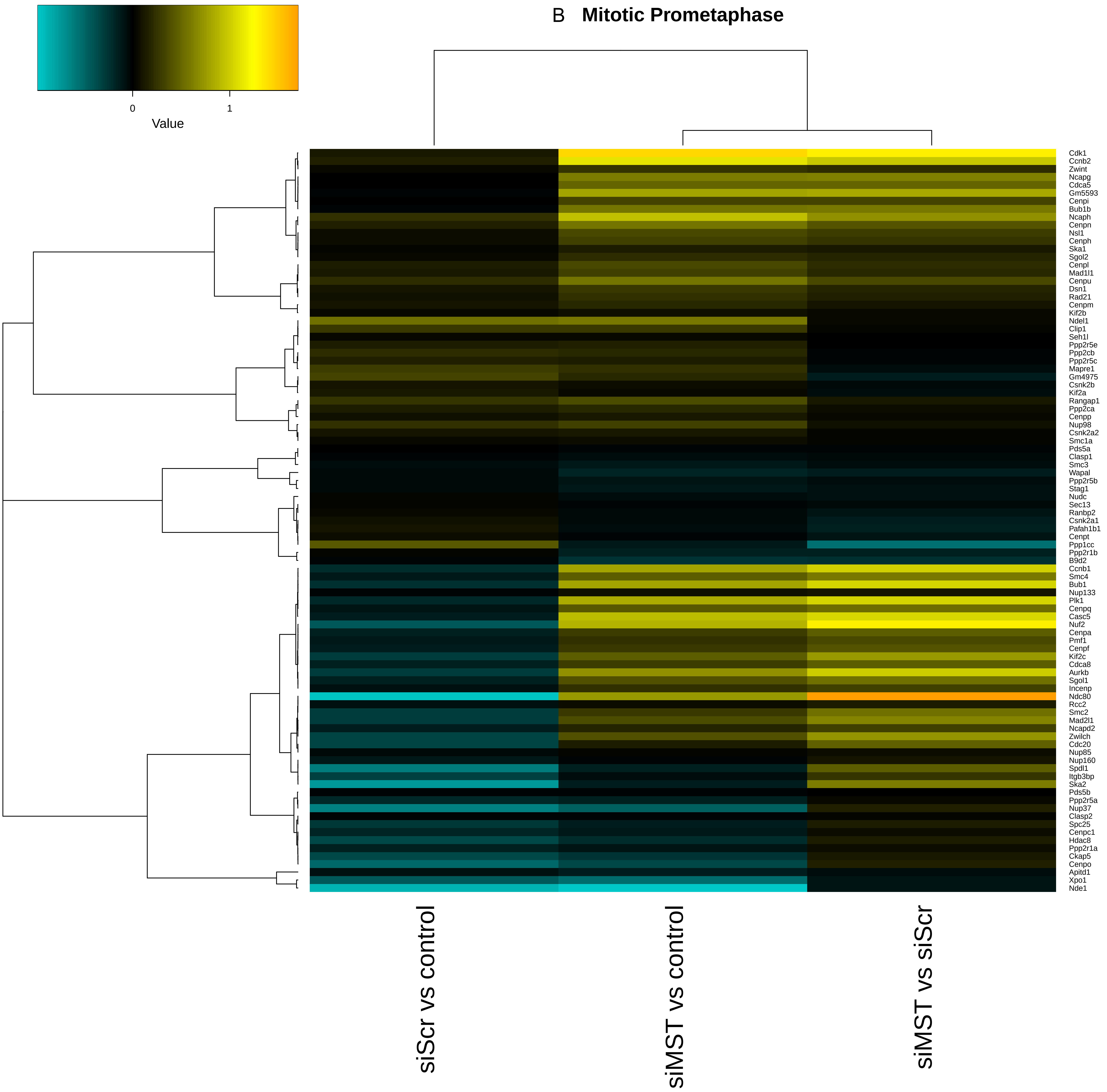
B Mitotic Prometaphase
Value
0
1
Cdk1
Ccnb2
Zwint
Ncapg
Cdca5
Gm5593
Cenpi
Bub1b
Ncaph
Cenpn
Nsl1
Cenph
Ska1
Sgol2
Cenpl
Mad1l1
Cenpu
Dsn1
Rad21
Cenpm
Kif2b
Ndel1
Clip1
Seh1l
Ppp2r5e
Ppp2cb
Ppp2r5c
Mapre1
Gm4975
Csnk2b
Kif2a
Rangap1
Ppp2ca
Cenpp
Nup98
Csnk2a2
Smc1a
Pds5a
Clasp1
Smc3
Wapal
Ppp2r5b
Stag1
Nudc
Sec13
Ranbp2
Csnk2a1
Pafah1b1
Cenpt
Ppp1cc
Ppp2r1b
B9d2
Ccnb1
Smc4
Bub1
Nup133
Plk1
Cenpq
Casc5
Nuf2
Cenpa
Pmf1
Cenpf
Kif2c
Cdca8
Aurkb
Sgol1
Incenp
Ndc80
Rcc2
Smc2
Mad2l1
Ncapd2
Zwilch
Cdc20
Nup85
Nup160
Spdl1
Itgb3bp
Ska2
Pds5b
Ppp2r5a
Nup37
Clasp2
Spc25
Cenpc1
Hdac8
Ppp2r1a
Ckap5
Cenpo
Apitd1
Xpo1
Nde1
siScr vs control
siMST vs control
siMST vs siScr

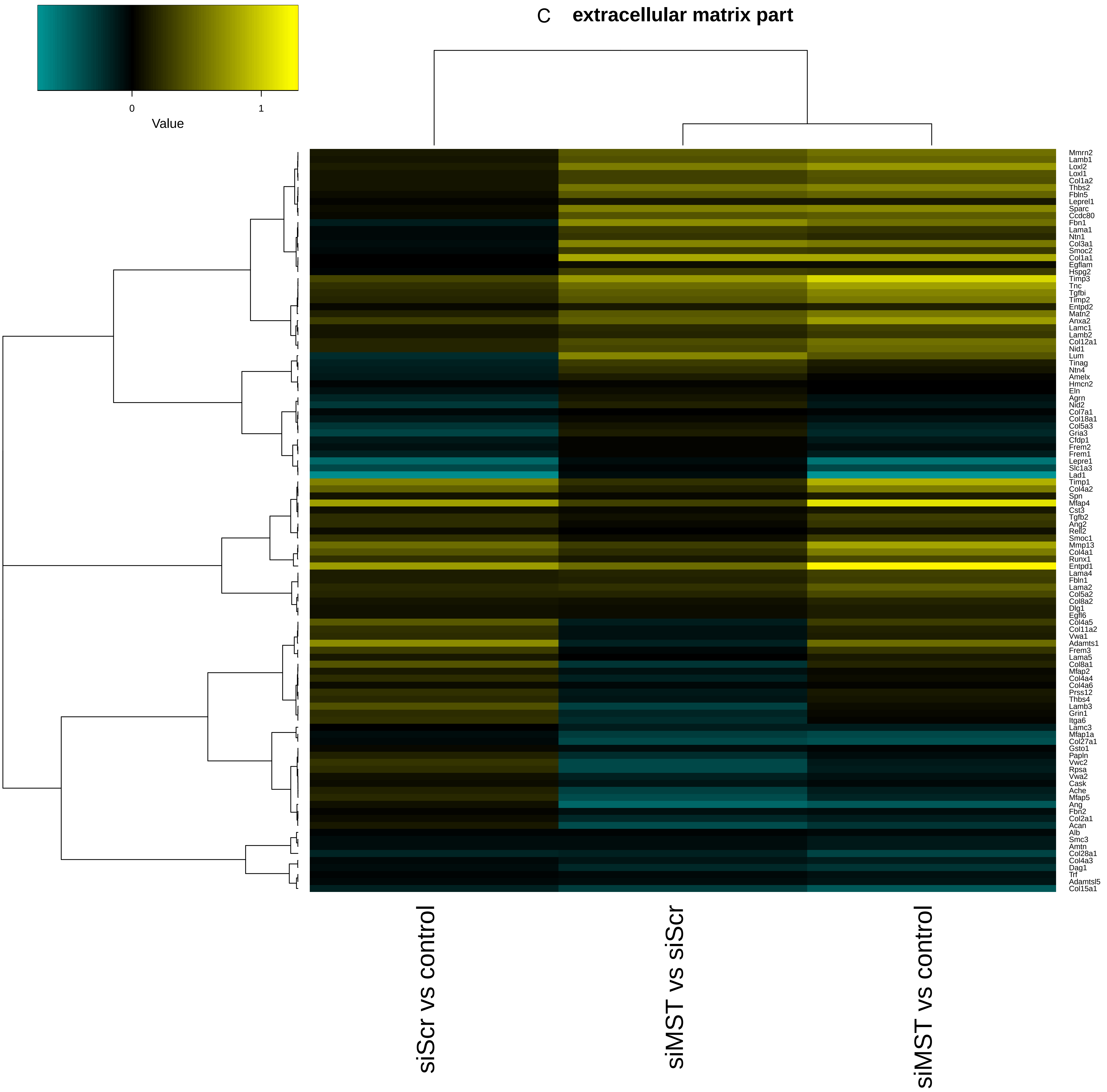
C extracellular matrix part
Value
0
1
Mmrn2
Lamb1
Loxl2
Loxl1
Col1a2
Thbs2
Fbln5
Leprel1
Sparc
Ccdc80
Fbn1
Lama1
Ntn1
Col3a1
Smoc2
Col1a1
Egflam
Hspg2
Timp3
Tnc
Tgfbi
Timp2
Entpd2
Matn2
Anxa2
Lamc1
Lamb2
Col12a1
Nid1
Lum
Tinag
Ntn4
Amelx
Hmcn2
Eln
Agrn
Nid2
Col7a1
Col18a1
Col5a3
Gria3
Cfdp1
Frem2
Frem1
Lepre1
Slc1a3
Lad1
Timp1
Col4a2
Spn
Mfap4
Cst3
Tgfb2
Ang2
Rell2
Smoc1
Mmp13
Col4a1
Runx1
Entpd1
Lama4
Fbln1
Lama2
Col5a2
Col8a2
Dlg1
Egfl6
Col4a5
Col11a2
Vwa1
Adamts1
Frem3
Lama5
Col8a1
Mfap2
Col4a4
Col4a6
Prss12
Thbs4
Lamb3
Grin1
Itga6
Lamc3
Mfap1a
Col27a1
Gsto1
Papln
Vwc2
Rpsa
Vwa2
Cask
Ache
Mfap5
Ang
Fbn2
Col2a1
Acan
Alb
Smc3
Amtn
Col28a1
Col4a3
Dag1
Trf
Adamtsl5
Col15a1
siScr vs control
siMST vs siScr
siMST vs control

**Figure S7– Heat maps of genes regulated by siMST compared to siScr**

A Interferon gamma signalling

B extracellular matrix remodelling

C mitotic prometaphase

Appendix Figure S8

a

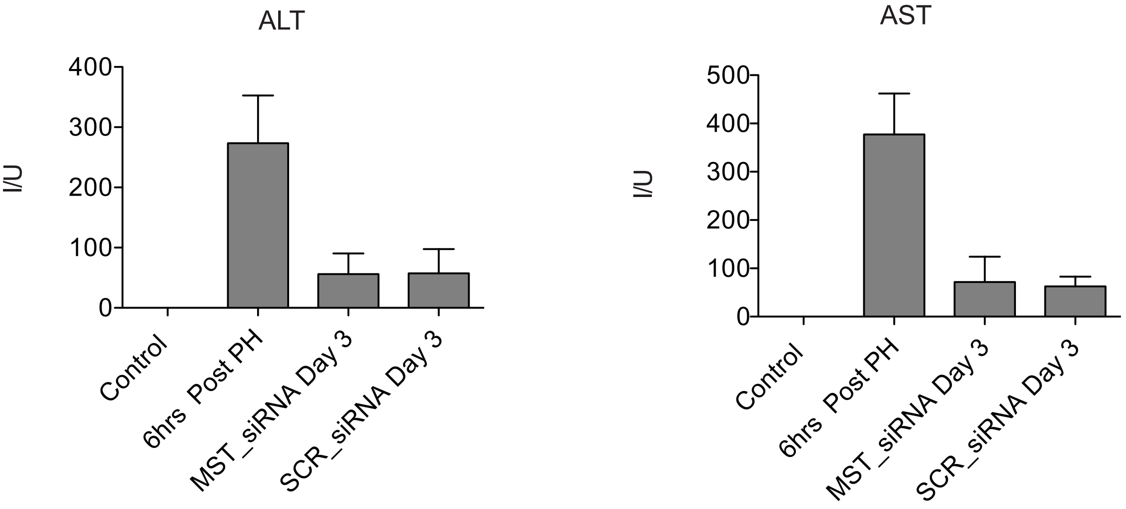


b

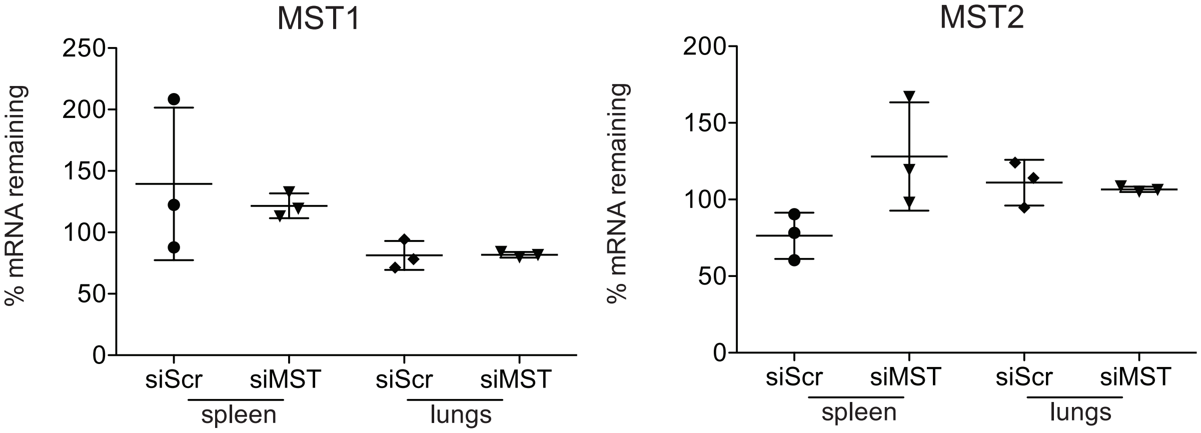


**Figure S8 Assessment of liver injury and off target effects from siRNA:liposomes**

A Assessment of liver injury by serum levels of AST and ALT 6 h post PH (positive control) and day 3 after injection of liposome coupled siMST or siScr siRNA.

B RT-qPCR of rmouse spleen and lung tissue after i.v injection of siScr or siMST liposome complex measured by RT-qPCR. Data are presented as mean ± SD. Student's t-test was used to calculate p values of siScr to siMST treated animals, with no significant alterations observed.

Appendix Figure S9

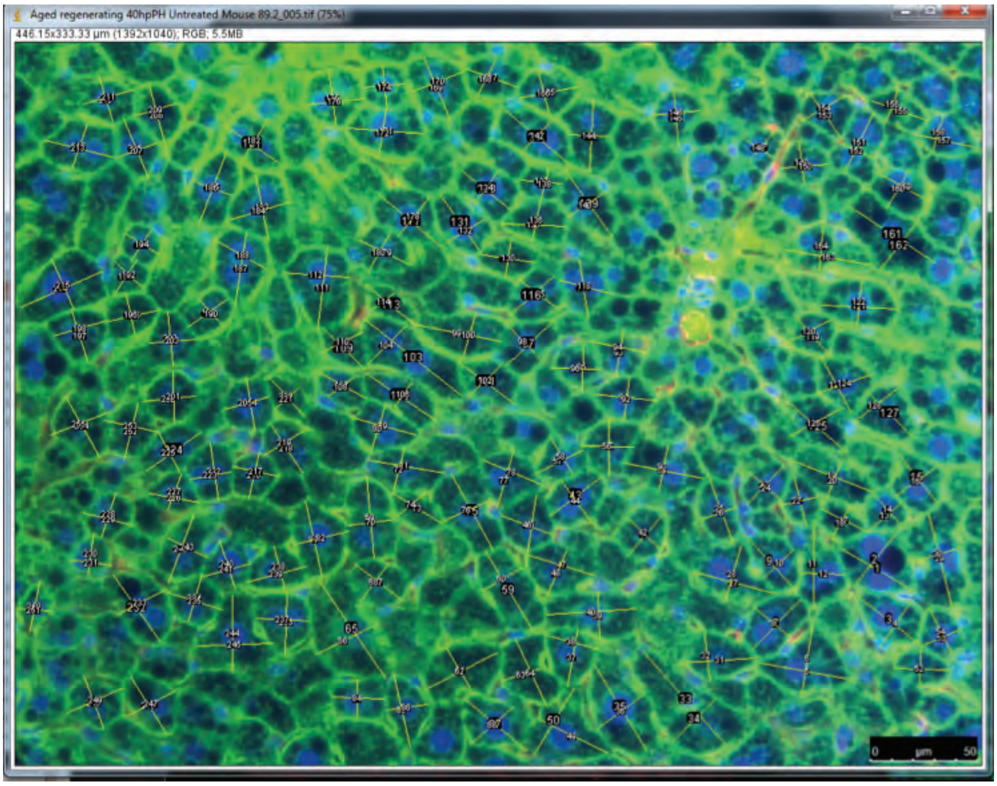


**Figure S9 Measurement of hepatocyte dimensions**

Representative photomicrograph of hepatocyte size calculation

Appendix Figure S10

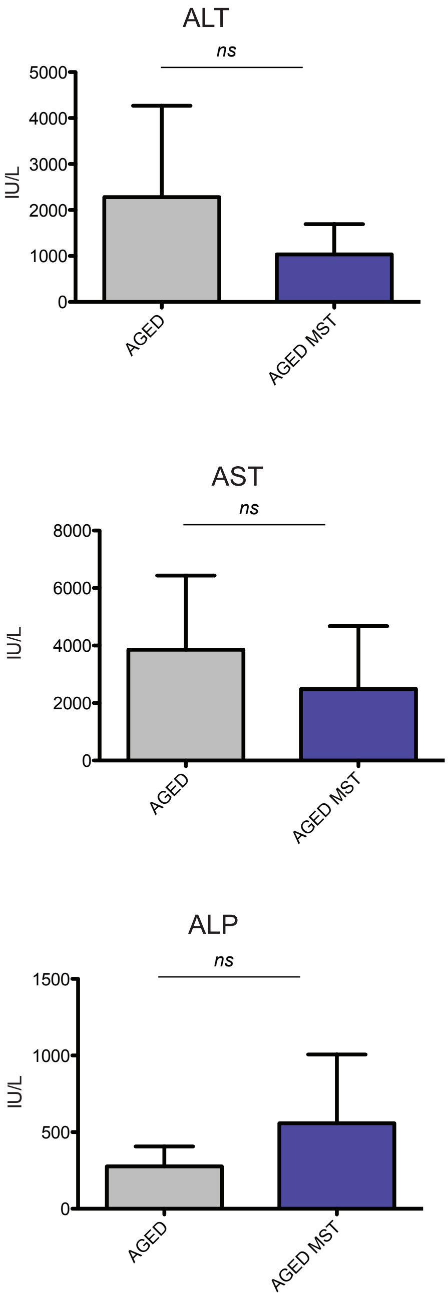


**Figure S10 Serum transaminases (ALT and AST) and alkaline phosphatase (ALP) in regenerating aged livers ± MST KD (aged MST)**

Data are represented as international units (IU) as measured in mice 40 h post PH in aged and aged + siMST KD mice. Bars are the mean ±SD, n=6 animals per group.
