# Supplementary material for: Impaired liver regeneration in aged mice can be rescued by silencing Hippo core kinases MST1 and MST2
Source: EMBO Mol Med. 2016 Dec 9;9(1):46–60. doi: 10.15252/emmm.201506089 (PMC5210079; doi:10.15252/emmm.201506089)
Supplement: Supplementary file 2 — Expanded View Figures PDF [file EMMM-9-46-s002.pdf]

Expanded View Figures

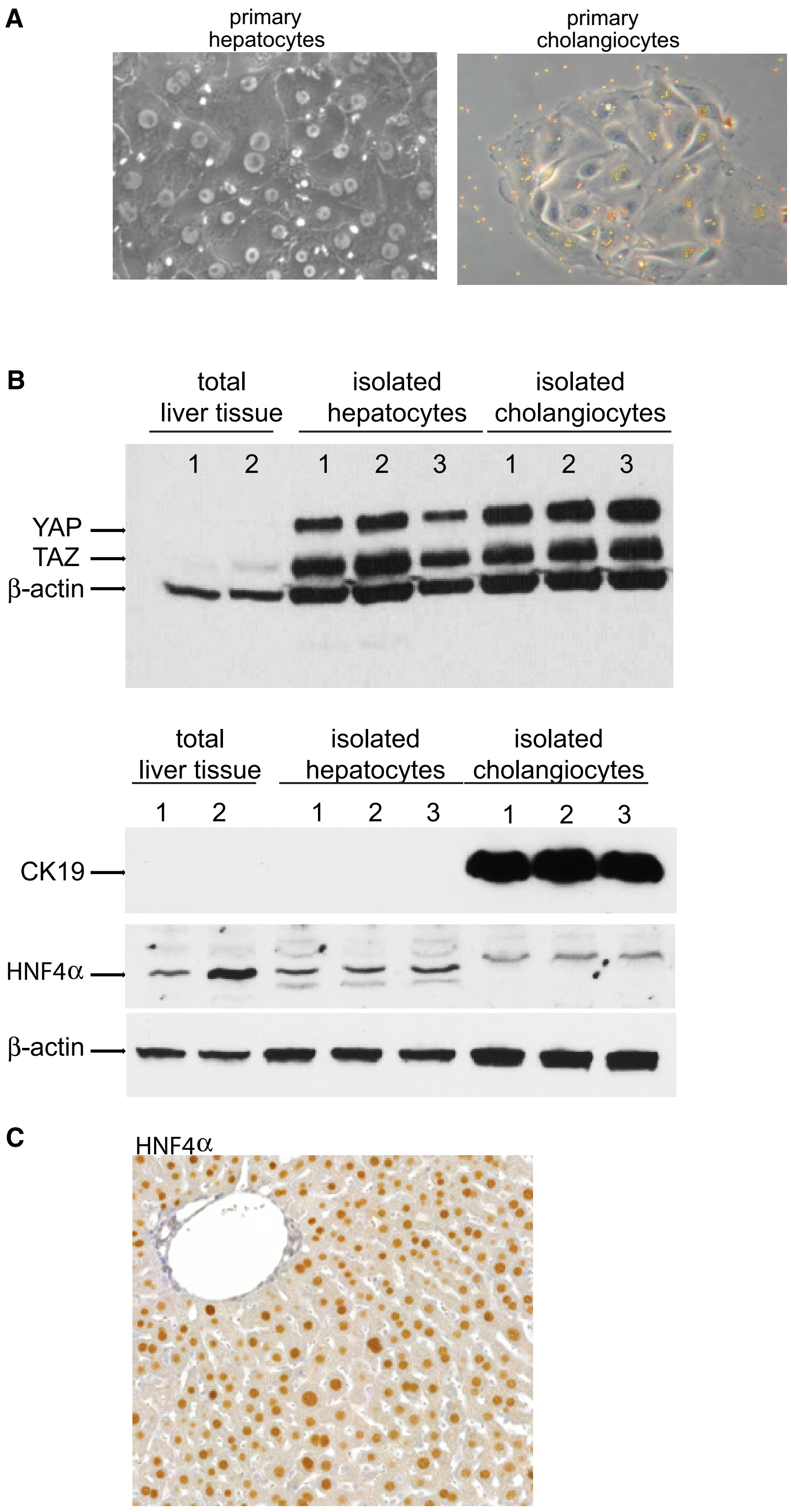

Figure EV1. YAP and TAZ expression in isolated hepatocytes and cholangiocytes.

A Photomicrographs of isolated hepatocytes (left panel) and cholangiocytes (right panel) in culture. Cholangiocytes (3 day post-isolation) are still bound with anti-CD326 (EpCam, HEA125) beads demonstrating specificity.

B Western blot detection of YAP and TAZ in proteins extracted from isolated hepatocytes and cholangiocytes and total liver tissue. Antibodies against CK19 and HNF4α were used to confirm purity of the populations and β-actin was used as a loading control. Representative results from a single experiment with $n = 3$ independent cell isolations and $n = 2$ total liver tissues.

C Photomicrograph of mouse liver tissue immunostained with an antibody against HNF4α (used in panel B) to demonstrate hepatocyte specificity.

Source data are available online for this figure.

 © 2016 The Authors

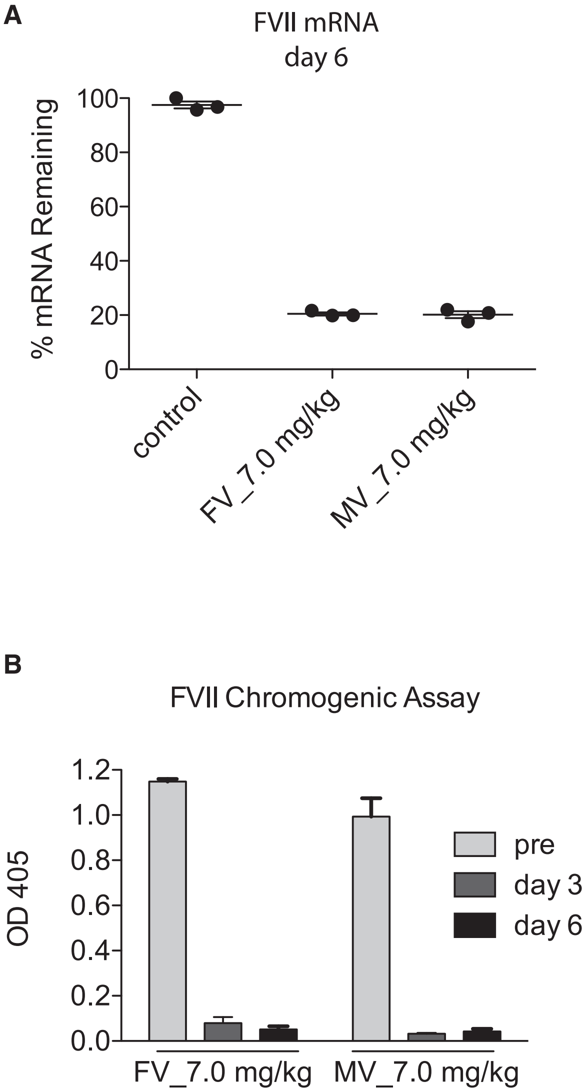

Figure EV2. Verification of *in vivo* efficacy of siRNA targeting of hepatocytes.

siRNA–liposome complex efficiency in targeting the hepatocytes was assessed by depleting the coagulator factor FVII which is exclusively produced by the hepatocyte population. siRNA targeting FVII was injected either by femoral vein (FV) or mesenteric vein (MV) at a concentration of 7.0 mg/kg.

A FVII % mRNA remaining analysed by RT–qPCR on day 6 post-FV or post-MV siRNA-FVII: liposome complexes injection.

B FVII protein levels measured in the blood of mice after injection of siRNA-FVII: liposome complexes by a chromogenic assay.

Data information: Representative results from two independent experiments with n = 3 animals per group. Each dot is representative for a single animal and the groups/columns are representative of the mean ± SD.

© 2016 The Authors

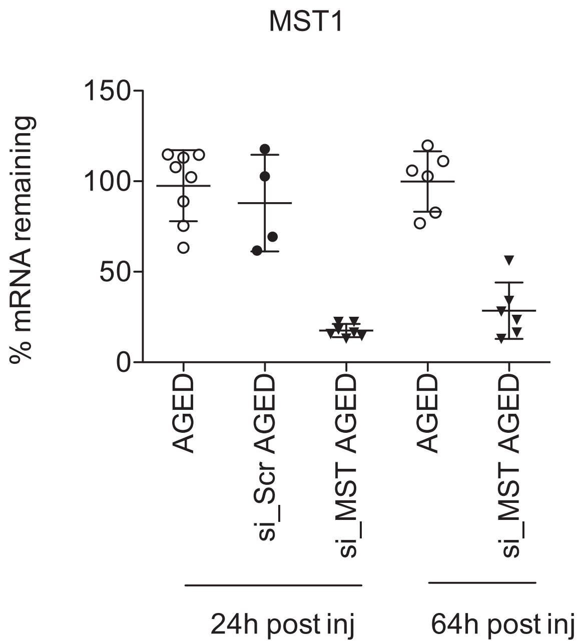

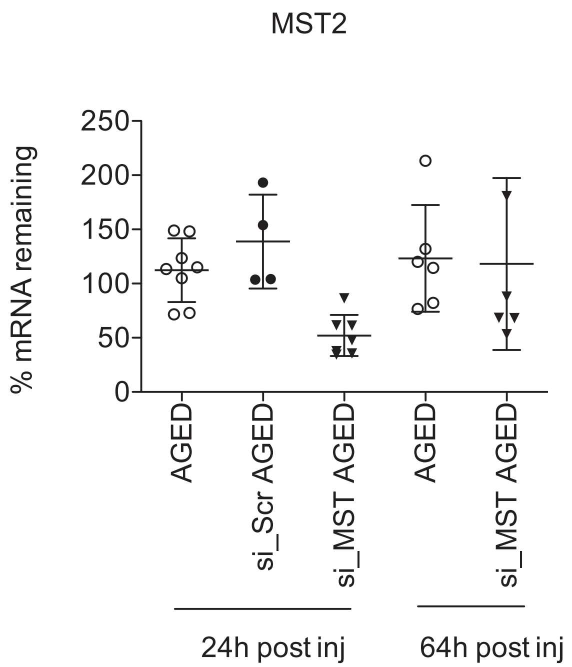

Figure EV3. Efficiency of siRNA targeting in aged livers.

Percentage of *Mst1* and *Mst2* mRNA remaining in aged mice liver 24 h (24 h pre-PH + 0 h post-PH) and 64 h (injected 24 h pre-PH + 40 h post-PH) after venous injection of siScr or siMST liposome complex measured by RT–qPCR. Non-matched liver tissue from untreated aged mice was used as a control. Representative results from a single experiment with $n = 6$ animals for group. Each dot is representative for a single animal and the groups are representative of the mean ± SD.

 © 2016 The Authors
