# Supplementary figures and images for: Impaired liver regeneration in aged mice can be rescued by silencing Hippo core kinases MST1 and MST2
Source: EMBO Mol Med. 2016 Dec 9;9(1):46–60. doi: 10.15252/emmm.201506089 (PMC5210079; doi:10.15252/emmm.201506089)

Figure EV1

anti-YAP/TAZ and anti- $\beta$ -actin

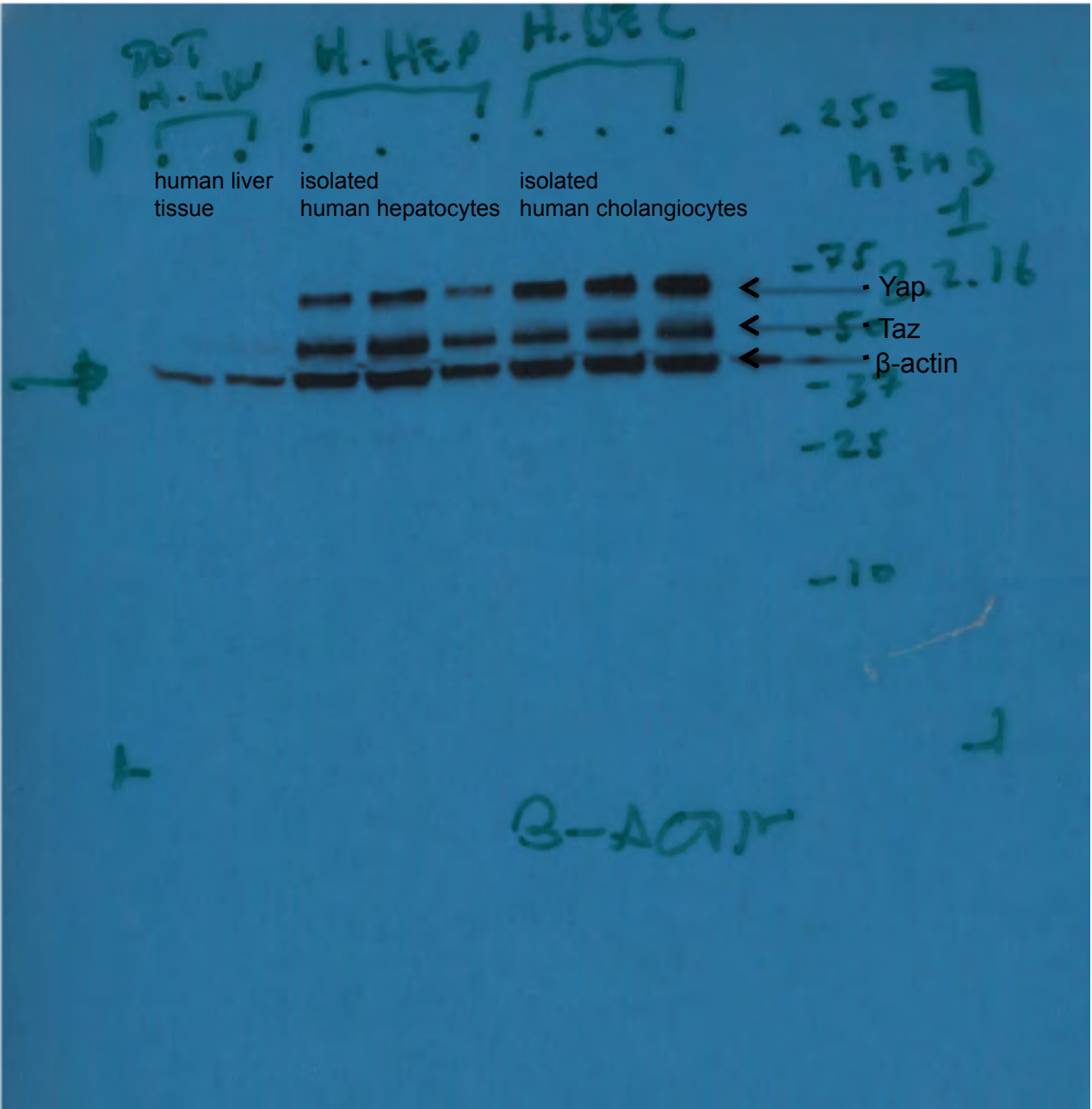

Figure EV1

anti-HNF-4 $\alpha$

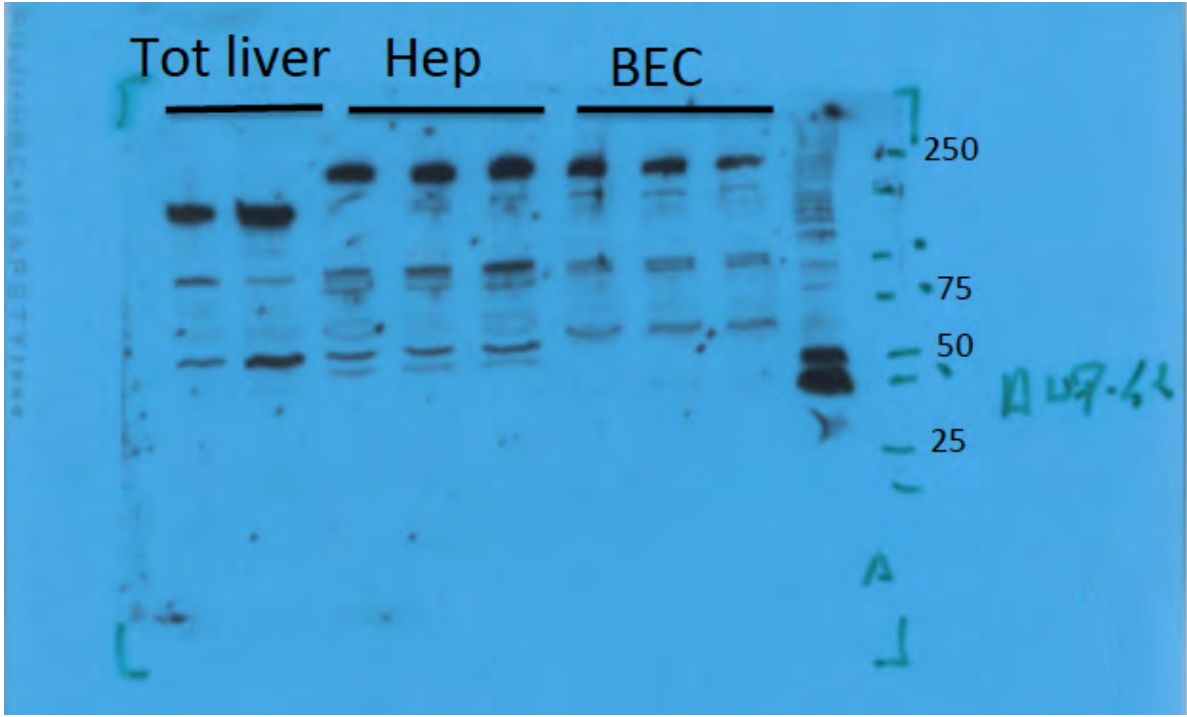

anti-CK19

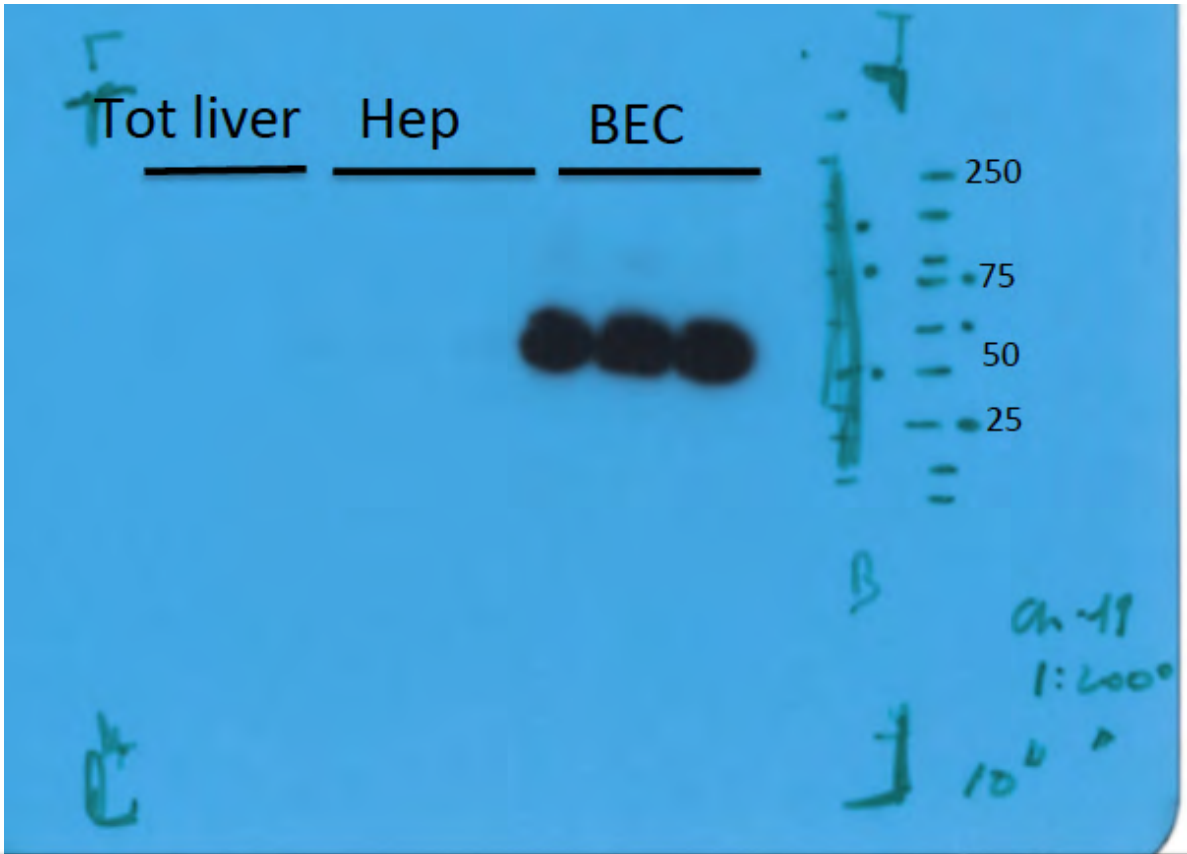

Supplement: Supplementary file 3 — Source Data for Expanded View [file EMMM-9-46-s006.zip › EMM_06089_EV_source_data/EMM_06089_EV_source_data/FigureEV1.pdf]

Figure 4B

anti-ph-LATS and anti-MST

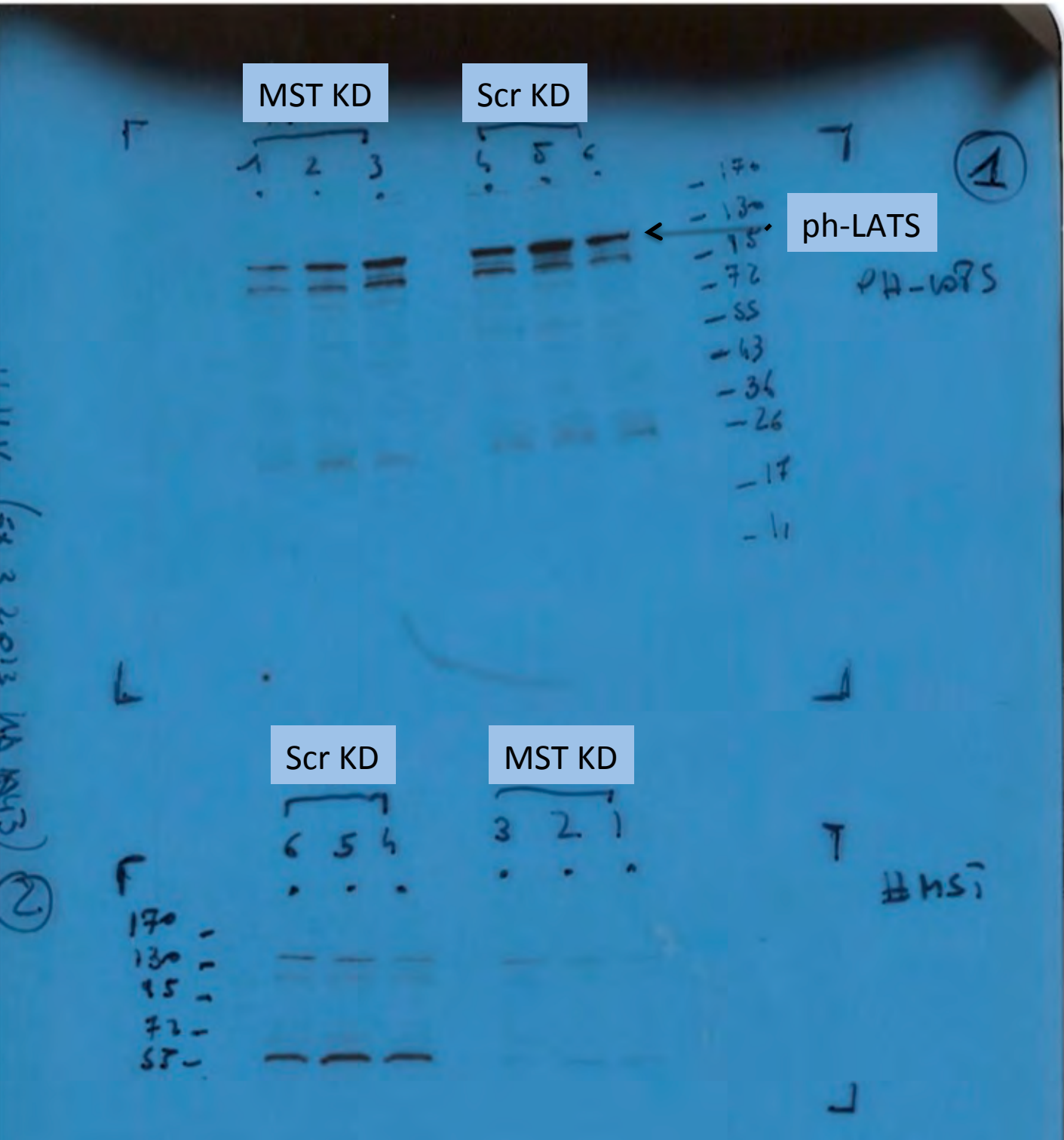

Figure 4B

anti-β-actin

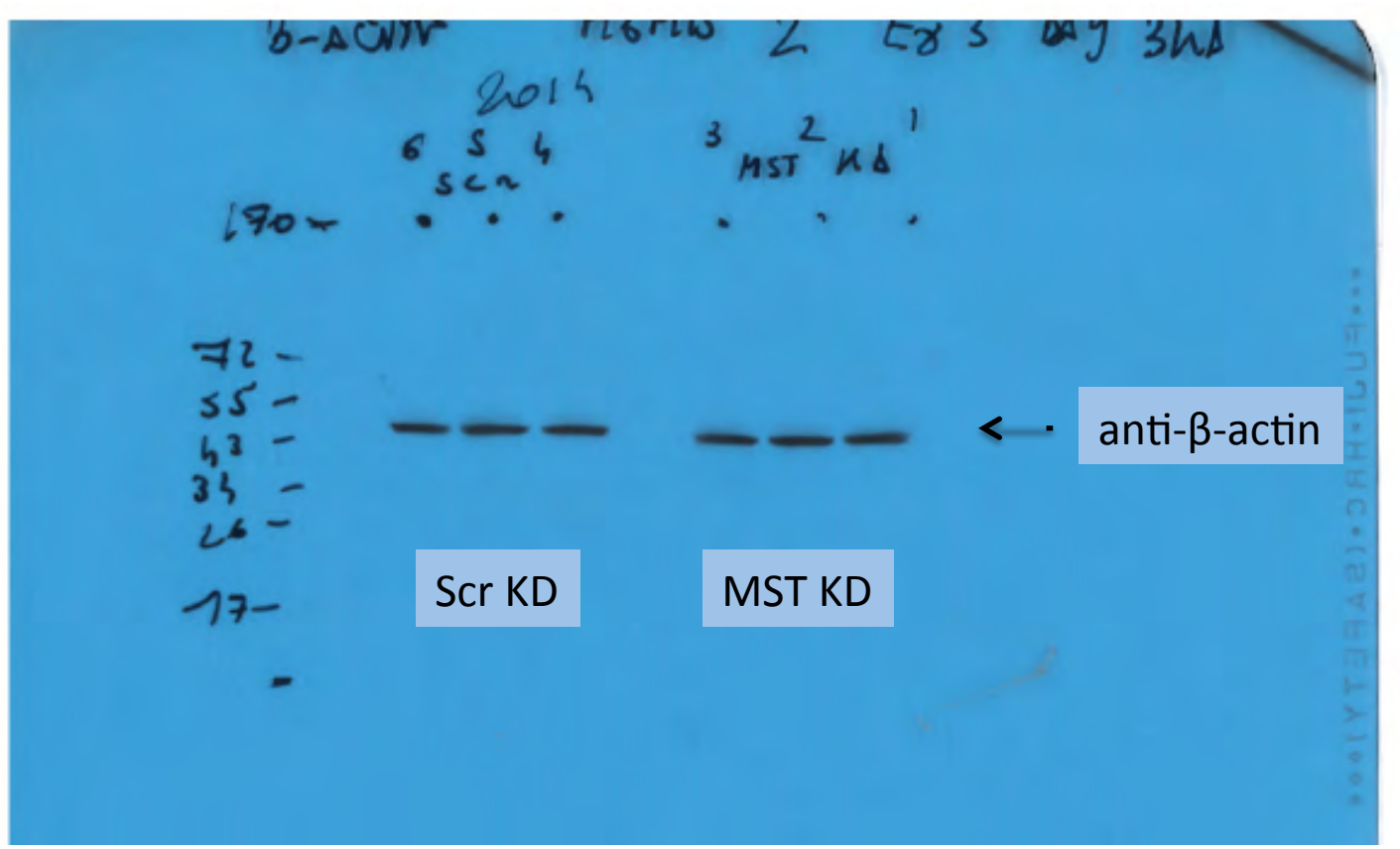

Supplement: Supplementary file 7 — Source Data for Figure 4 [file EMMM-9-46-s005.pdf]
