# Supplementary material for: Impaired liver regeneration in aged mice can be rescued by silencing Hippo core kinases MST1 and MST2
Source: EMBO Mol Med. 2016 Dec 9;9(1):46–60. doi: 10.15252/emmm.201506089 (PMC5210079; doi:10.15252/emmm.201506089)

Figure 1B

anti-p-MST

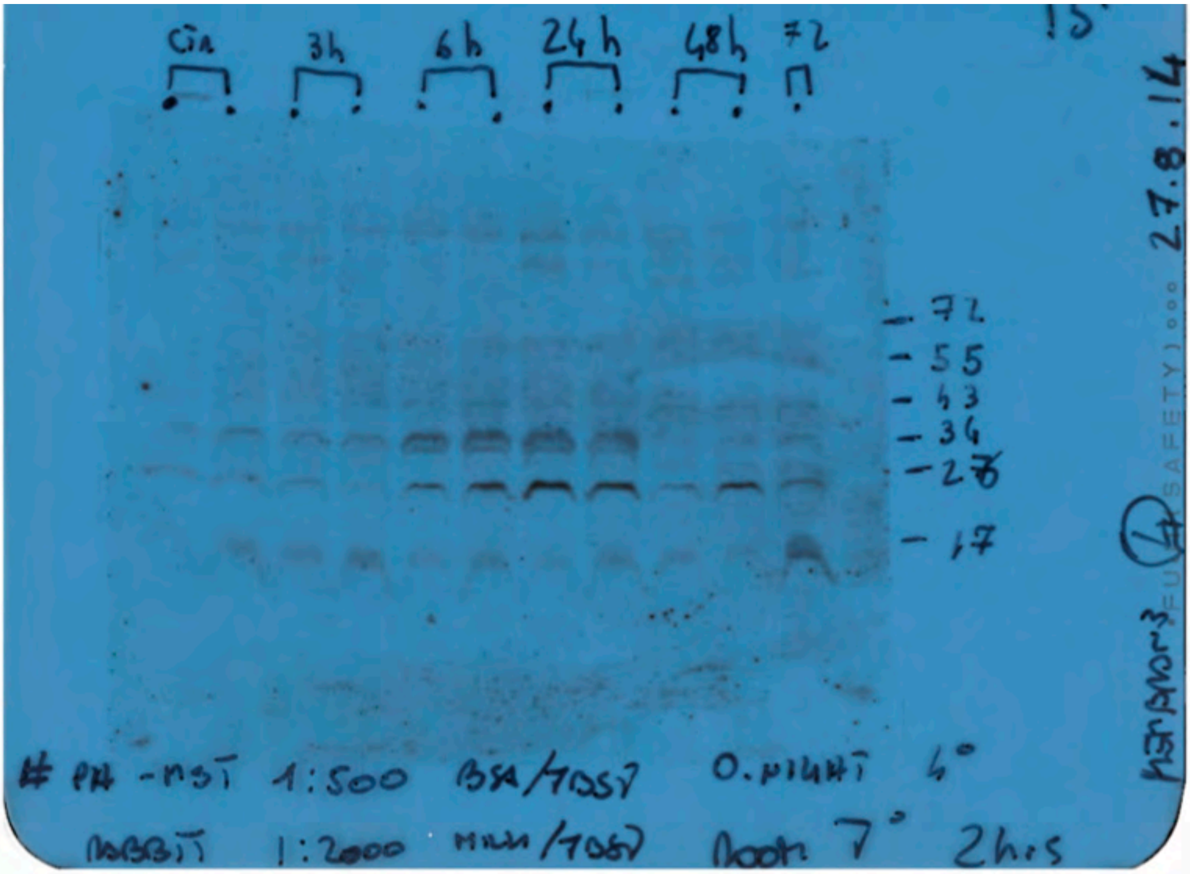

Figure 1B

anti-MST

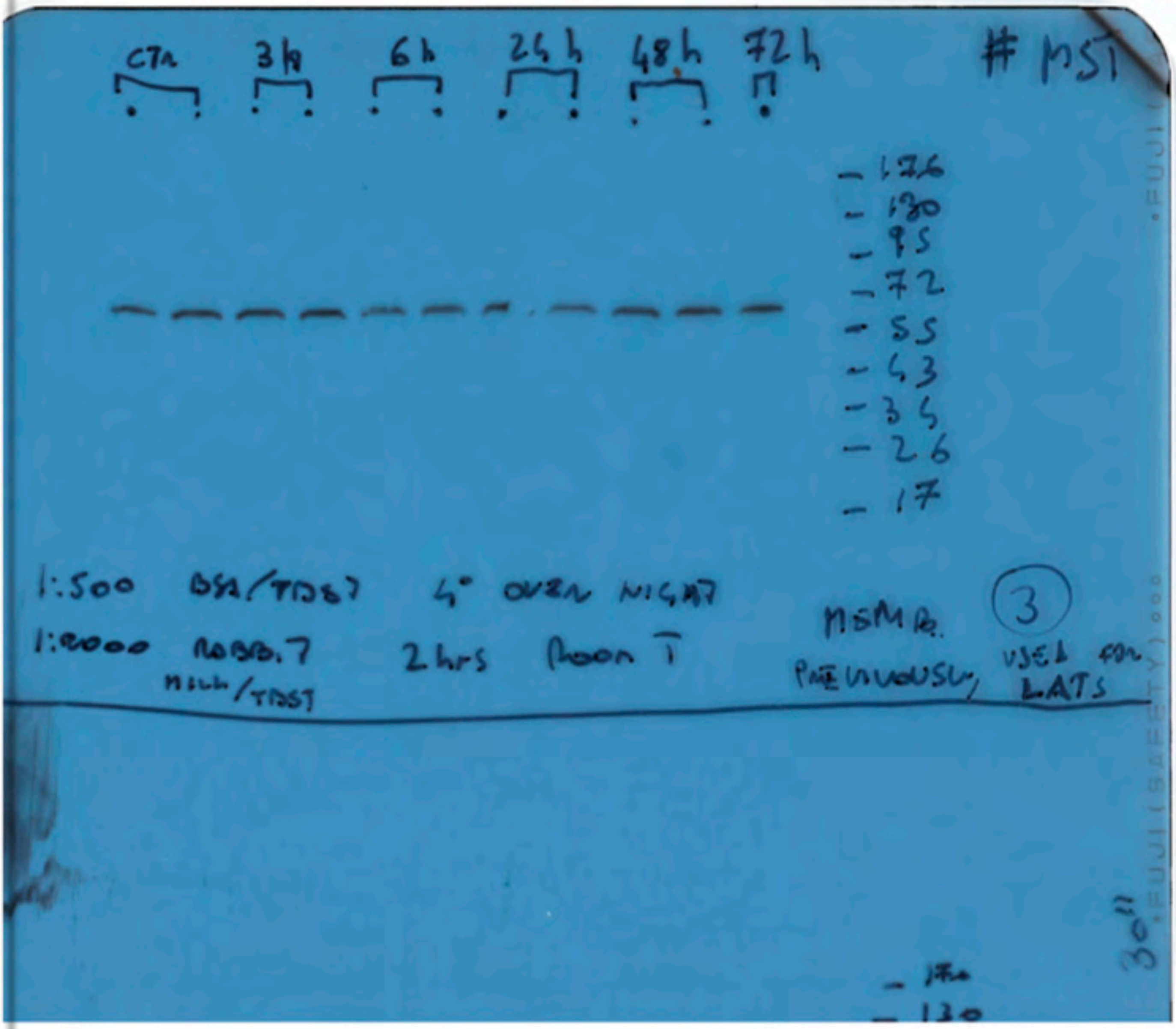

Figure 1B

anti-ph-LATS

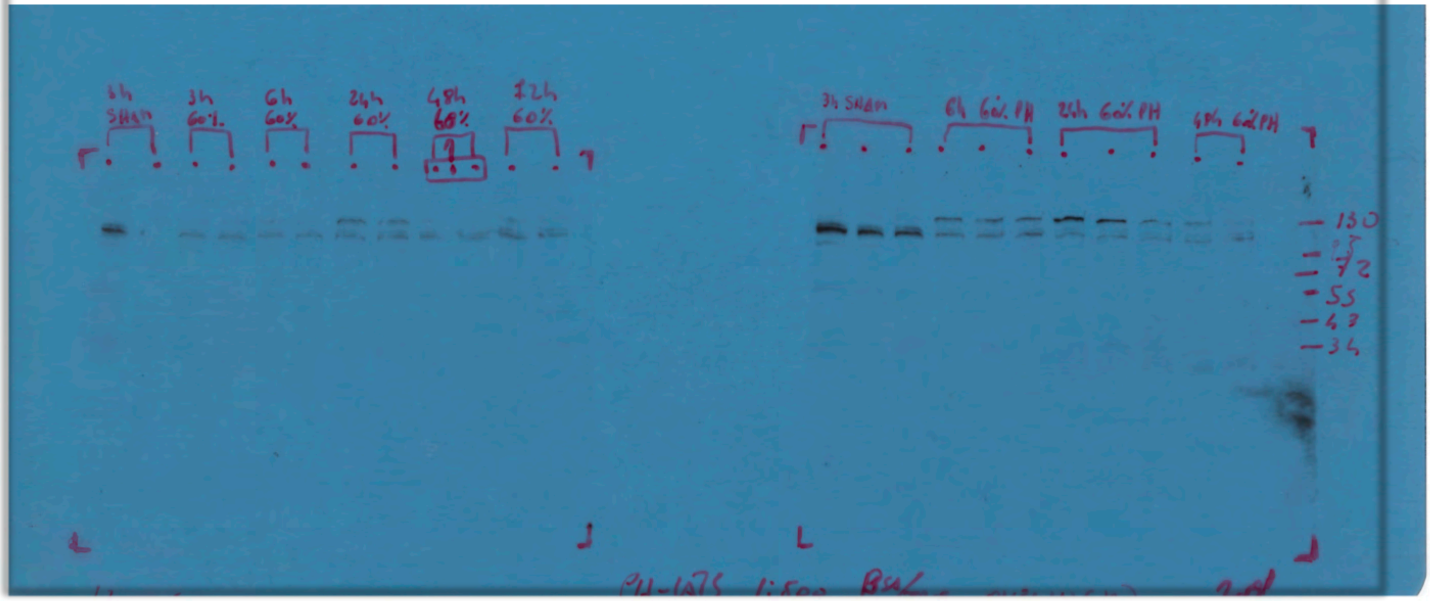

anti-LATS

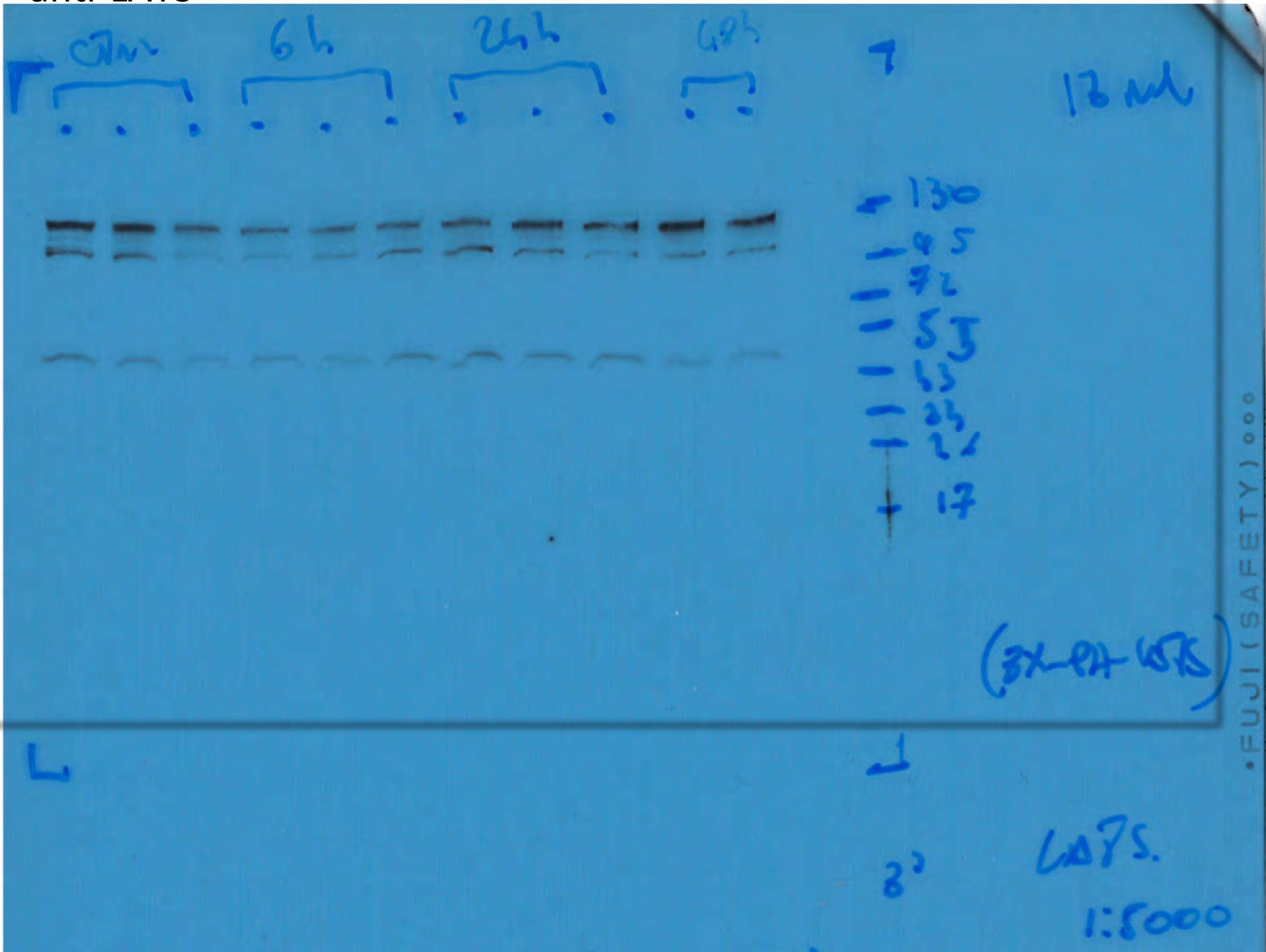

Figure 1B

anti-TBP

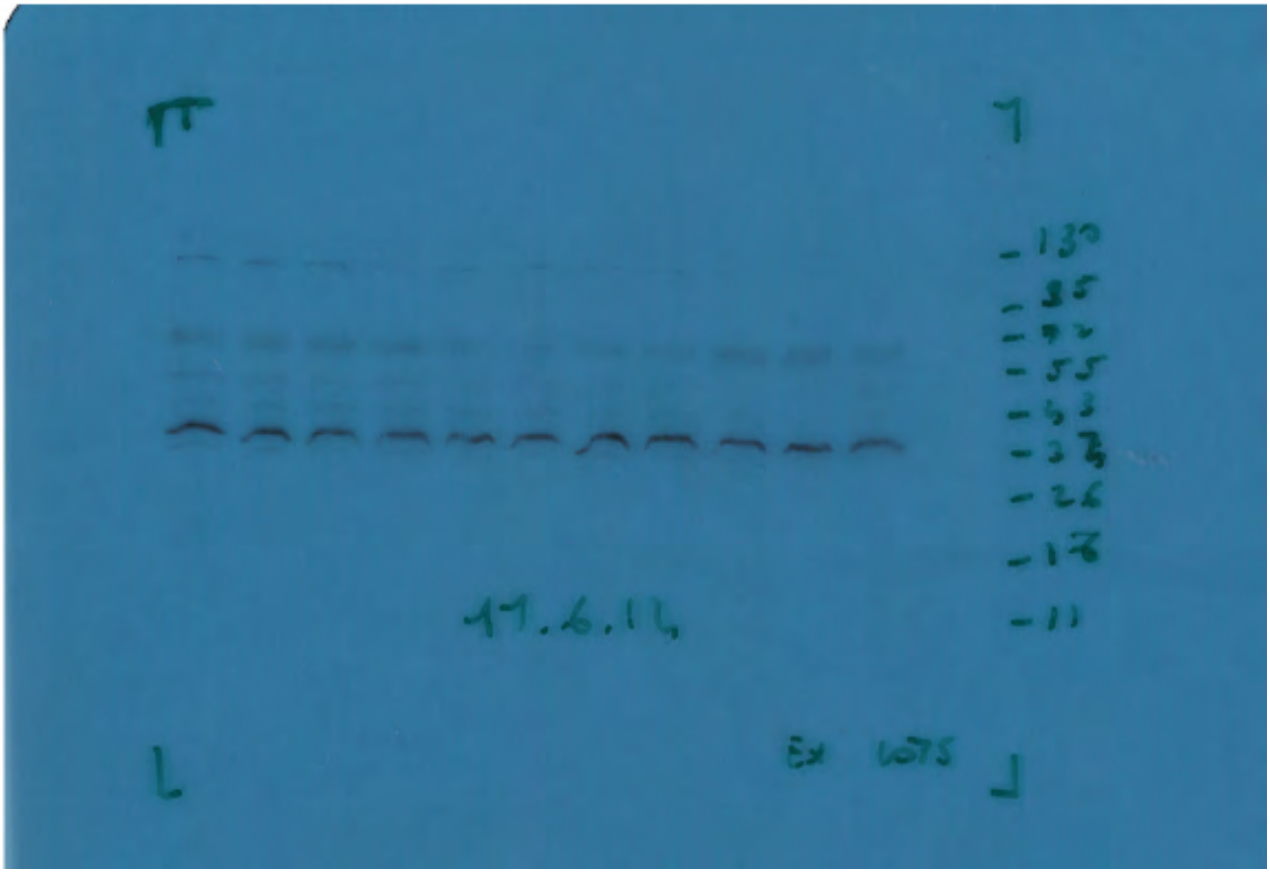

Figure 1C

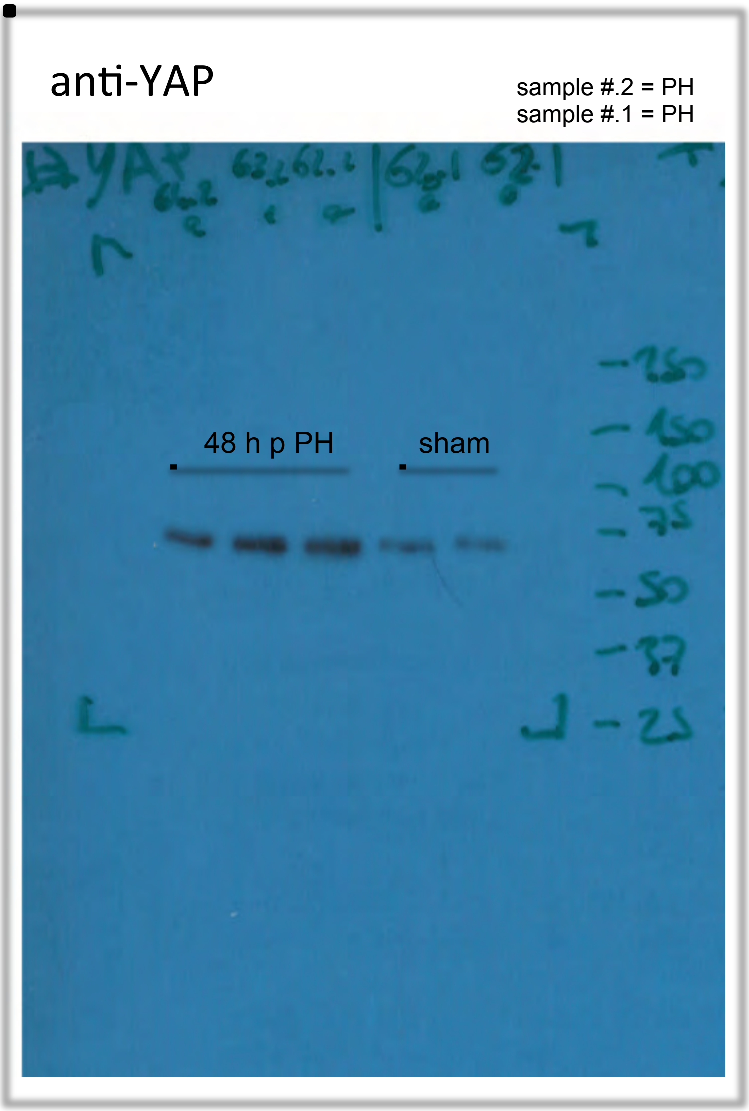

Figure 1E

anti-Yap/Taz

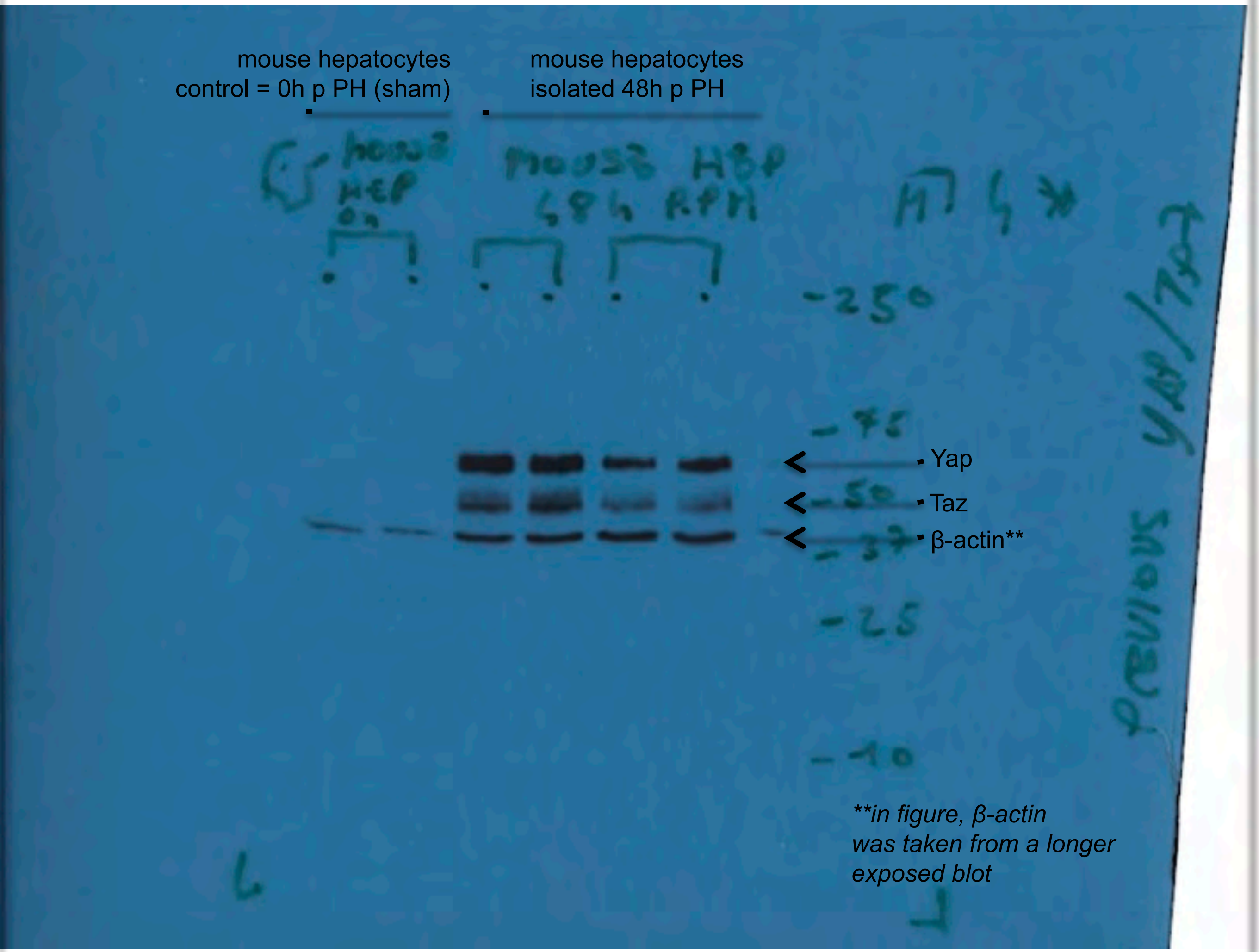

Supplement: Supplementary file 5 — Source Data for Figure 1 [file EMMM-9-46-s003.pdf]
