# Supplementary material for: Impaired liver regeneration in aged mice can be rescued by silencing Hippo core kinases MST1 and MST2
Source: EMBO Mol Med. 2016 Dec 9;9(1):46–60. doi: 10.15252/emmm.201506089 (PMC5210079; doi:10.15252/emmm.201506089)

Figure 3A

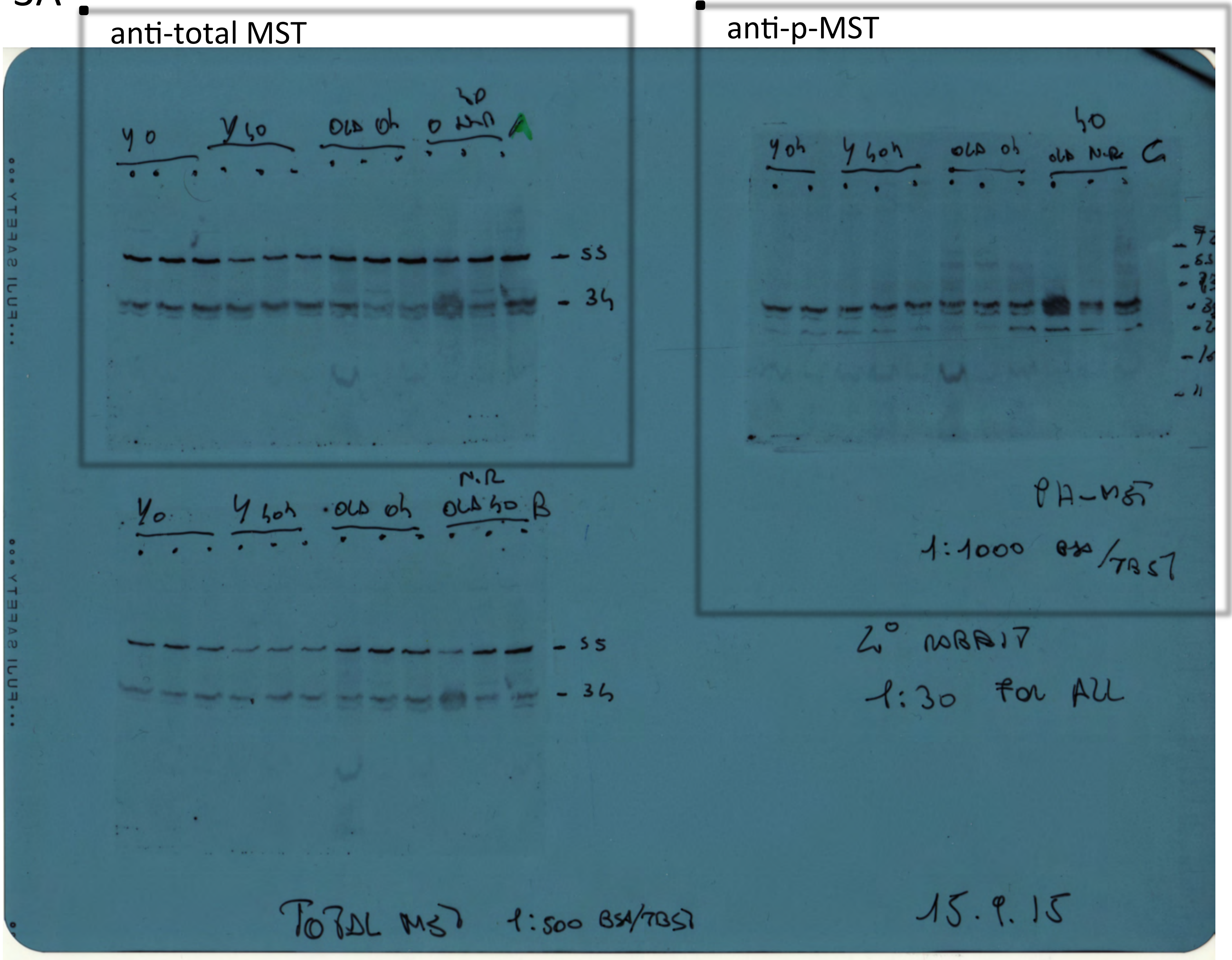
anti-total MST
anti-p-MST
55
34
1:1000 BSA/TBST
1:30 for ALL
TOTAL MST 1:500 BSA/TBST
15.9.15

Figure 3A

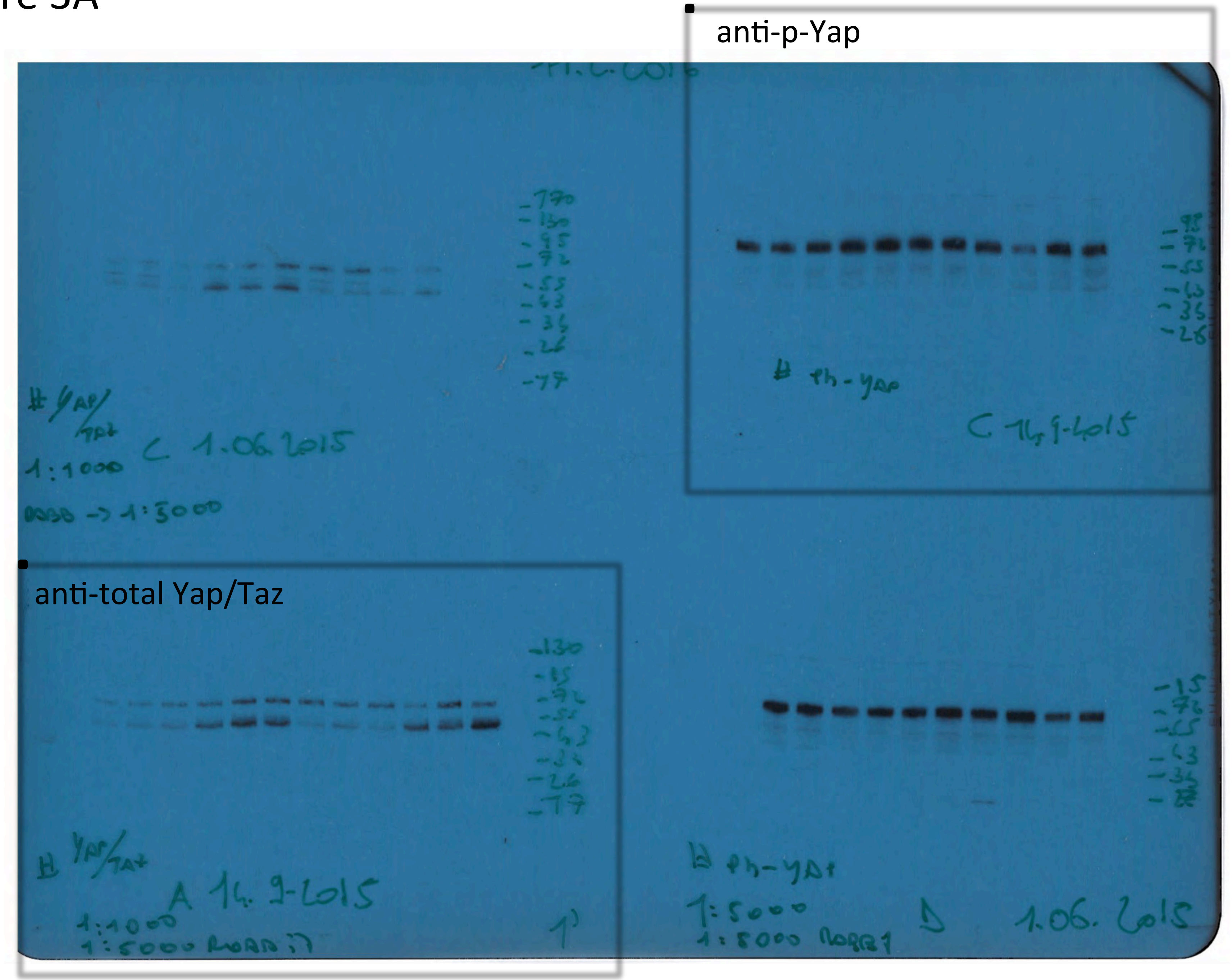

Figure 3A

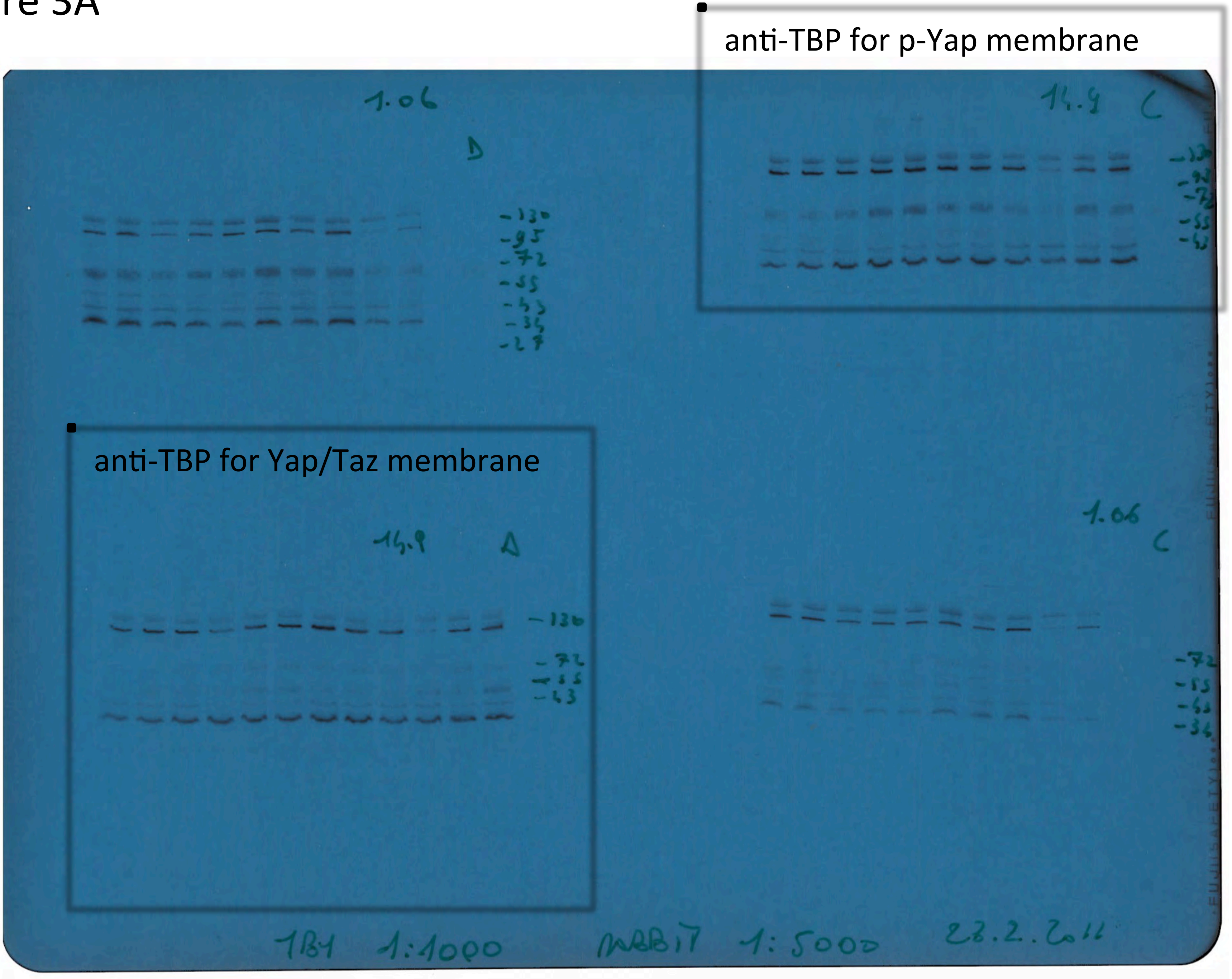

Supplement: Supplementary file 6 — Source Data for Figure 3 [file EMMM-9-46-s004.pdf]
